# Let’s Try Social Prescribing in Sweden (SPiS) – an Interventional Project Targeting Loneliness among Older Adults Using a Model for Integrated Care: A Research Protocol

**DOI:** 10.5334/ijic.5609

**Published:** 2021-06-22

**Authors:** Erika Johansson, Frida Jonsson, Emil Rapo, Anna-Sofia Lundgren, Åsa Hörnsten, Ingeborg Nilsson

**Affiliations:** 1 Umeå University, SE

**Keywords:** social prescribing, loneliness, social participation, integrated care, complex intervention, realist evaluation

## Abstract

**Introduction:**

Loneliness and social isolation among older adults (≥65) are an emerging issue of public concern, associated with increased morbidity and mortality. Today there is no systematic intervention developed, implemented or evaluated in Sweden addressing loneliness. The overall aim for this project is to develop, test and refine a person-centred Swedish model for social prescribing (SPiS), and to assess whether and how it reduces loneliness, promotes health and improves well-being among older adults.

**Description:**

The focus will be to develop, culturally adapt, evaluate and refine the SPiS model. Following the sequential structure of realist evaluation in three consecutive phases qualitative and quantitative data along with subsequent analysis methods will be collected and utilized. The project will provide knowledge of what works with the social prescribing model, for whom, in what conditions and why, in relation to loneliness, health and well-being among older adults.

**Discussion:**

SPiS has the unique position of providing initial knowledge regarding how to reduce loneliness in the Swedish context. However, evaluation is complex as this research goes beyond the unidimensional question “Is it working?”.

**Conclusion:**

Developing, implementing and evaluating such a complex program needs systematic and close evaluation.

## Introduction

In order for society to meet the health challenges and the complex care needs of the future, it is essential to strive for development of new innovative treatment models. In Sweden, there is no single model for integrated care with the aim of closing the gap between primary health care and community based rehabilitation. Health services are still focused on and designed to cure acute conditions or symptoms and tend to manage health issues in disconnected and fragmented ways, without a holistic understanding of health. In contrary to what has been stated in policy documents [1], they lack coordination across care providers, settings and time. Lack of coordination increases the risk of structural discrimination as it undermines the ability of older adults to have their voice heard and their needs met regardless of their human rights [2]. Increasing proportion of older adults poses challenges for the welfare states to facilitate integrated care developing new innovative areas for practice [3]. Healthcare costs are expected to increase dramatically, and will require innovative ways, with focus on quality of care as well as sustainability, to meet and prevent the ensuing complex needs among the older population [4].

Loneliness among older adults has become an issue of public concern. There is a high and stable prevalence of reported loneliness, with as many as half of older adults reporting serious or moderate loneliness [3]. In addition, in these pandemic times, social isolation increases the urgent need for action. There is also growing evidence on the significant health consequences in older adults e.g., systematic reviews show that social isolation and feelings of loneliness among older adults negatively affect physical and mental health [5]. Deterioration in physical and mental health are especially prevalent in older people with health problems, and are associated with social and socio-demographic aspects such as gender, civil status and social networks [6].

Nevertheless, loneliness should not be considered as a fixed condition which cannot be changed. As well as the fact that loneliness affects the experience of health and well-being, social engagement can have a positive impact [7,8]. Instead research has demonstrated the importance of social relations and social engagement for older people’s health, well-being and cognitive health. Important criteria for the experience of well-being [9] are feelings of belonging and connectedness to family, friends and society. Social inclusion in society and strong social relationships may also decrease mortality rate; a meta-analysis of 148 longitudinal studies revealed a 50% reduction among people with strong social relationships [10]. In summary, research highlights essential factors that include social activities as protective factors in therapy methods, which confront rising isolation and subjective loneliness [11,12].

### Social Prescribing (SP)

Social engagement is frequently desired among older adults [13,14] and has a positive relation to self-rated health among older adults [15]. As such, there is a need to develop and evaluate person-centred interventions, which include support for social participation and which strive to prevent loneliness[16]. In 2002 the United Kingdom thus initiated Social Prescribing [SP], that focused on linking patients in primary care with sources of social engagement within their local community [17]. SP is one model for integrated care and entails expanding the options available to General Practitioners [GP] and other front line medical health staff, forwarding a client to existing social engagement [social activities] in the community that meet psychosocial needs and might affect loneliness [18]. Although, there are no standards in its procedures [17], SP provides the option of using a non-medical referral, either as stand-alone, or combined with existing treatments [19]. The activities provided in the local community for social engagement can be either in the volunteer or community sectors [20]. Challenges that have been described include the multiplicity of options and the logistical difficulties. Although the idea is simple, the implementation of the model is complex [18,21]. Essential for the intervention is that there is a direct referral from primary care, and an identified coordinator (link worker) who connects the person to local activities that meet their needs and desires [22].

Social Prescribing is currently being advocated and implemented, particularly in the UK. On the one hand, impact from SP has shown improvement in well-being, decrease of mental and physical symptoms, as well as potential benefit on social isolation and loneliness [23]. Further, studies on SP also show that social prescribing is potentially supportive to reduce demand on primary and secondary care, and has the possibility of delivering cost savings [24]. On the other hand, recent research also shows inconsistency and ambiguity in the effects of SP. Which calls for attention that provides sufficient determination of the model’s capacity [16,19].

Notwithstanding the inconclusiveness in research findings, there is also reason to delve into the possibilities of providing social prescribing as one approach of integrated care services in Sweden, and research the intervention’s potentials to reduce feelings of loneliness among the older generation. However, complex interventions that comprise multiple interacting components such as SP are challenging to evaluate, sensitive to context, and may not be easily transferred between national contexts. Challenges often described include the content and standardization of the intervention, the impact on the people involved (staff and patients), the organizational context of implementation [25], the development of outcome measures, and evaluations [16,21,26]. Rigorous and high quality research must thus be carried out including in the Swedish context.

### Introducing social prescribing in the Swedish context

Since complex interventions that comprise multiple interacting components such as SP may be challenging to evaluate as well as sensitive to and not easily transferred between national settings, there is a need to culturally adapt the UK version of SP to ensure contextual fit in Sweden. In the current project, this process started with transferring the social prescribing model, as used in the UK, to a health clinic in the north of Sweden and in dialogue with the health care practitioner at the clinic a novel integrated care programme for Social Prescribing in Sweden (SPiS)was mapped out by the researchers and approved as a base for further development in the Swedish healthcare centre. The SPiS programme in its novel model constitutes the following procedure: I) identification of loneliness in the primary healthcare centre, II) referral to a specialised team for examination and assessment, III) individually adapted prescription of social activities through an in-depth person-centred interview where interests and preferences are matched with the local variety of social activities and prescribed, IV) support for engagement according to prescription in social activities in the local community and V) follow up at the primary healthcare centre. Hence, in order to meet the minimum requirements for quality in method development, this informal and unstructured process of knowledge building will require a more formal and structured process for theory development, testing and refinement of the SPiS which rationalizes the subsequent described project.

### Objectives

The overall aim for this project is thus to develop, test and refine a model for Social Prescribing in Sweden (SPiS), and assess whether and how it reduces loneliness, promotes health and improves well-being among older adults (≥65). Until now, social prescribing has not been adapted for and assessed in a Swedish context. Building upon the introductory work conducted so far and the sequential structure of the realist evaluation methodology the project will be divided into three consecutive phases. To facilitate this scope, the project will follow the realist evaluation methodology [27] to address the following general research questions:

1. What will a culturally adapted social prescribing model look like in the Swedish context, and how and under what circumstances is it expected to reduce loneliness, promote health and improve well-being among older people?
2. How do the practitioners and older adults experience trying the social prescribing model?
3. What works in the social prescribing model to reduce loneliness, promote health and improve the well-being of older adults; for whom, in what conditions and why?

### Conceptual and methodological framework

Our point of departure in this project is that the translation and implementation of SP into a Swedish context requires cultural adaptation. Firstly, it needs to be adapted to the structures and routines of the Swedish welfare system, which differs from the UK in various aspects, e.g., regarding how society is structured when it comes to perceived responsibilities of state, family and local community, and who delivers activities [28]. This also includes questions about who might be considered for this type of intervention, what professionals in the public sector should be given the opportunity to refer patients, and which social activities may be appropriate and for whom. Secondly, to arrive at an effective practice of referral, the programme also has to be adapted to the possibly different conditions for approaching a subject of loneliness within the meeting between the healthcare and the patient, person-centeredness.

To capture the different perspectives outlined above and ultimately attain an enriched understanding about the workings of social prescribing in the Swedish context, a theory-driven realist evaluation approach will constitute the methodological base of the project [27]. With roots in scientific realism and the work of Pawson and Tilley, realist evaluation extends beyond traditional efficacy assessments and the question of “does it work” by focusing instead – in sequential steps – on how, for whom, in what conditions and why the intervention is a] presumed to work, and b] whether [or not] it functions as intended [29]. In this regard, not only is this methodology suitable to assess complex interventions and their implementation contexts, but it is also useful in revealing assumptions about what ‘might cause change’ that are inherent but are seldom made explicit when the intervention is designed and implemented [27].

Undertaking a realist evaluation thus follows a cyclic process where the *programme theory* behind the intervention (SPiS in our case) and its implementation is first elicited in close collaboration with stakeholders who most likely have knowledge or thoughts about how the intervention might become successful [30]. In a second phase, the programme theory is then tested during the implementation in a primary healthcare centre. The experiences of the implementation are collected through qualitative and quantitative methods allowing the assumptions upon which the initial programme theory was based to be confirmed and/or rejected. This means that extensive amounts of data capturing aspects related, for example to the intervention and its impact or outcomes, processes, implementation, context and mechanisms need to be gathered – preferably from different, complementary sources. Based on the idea that interventions contribute to certain outcomes by influencing the behaviour or reasoning [i.e. mechanisms] of targeted actors in a particular context, the collected material is then synthesized in a third phase [31] Based on this analysis, the programme theory is then refined in the shape of a middle-range theory to establish whether [or not] the interventions work as intended and in that case, for whom, in what conditions and why [29].

An ethnographic approach [32] will also be employed to further increase our understanding about the cultural assumptions at play when the social prescribing programme is designed, implemented and refined. Ethnography generally refers to the critical quest of knowing the field of study from the perspective of its inhabitants and social relations. Moreover, it also, discusses the taken-for-granted assumptions upon which such perspectives are based [32,33]. It has been recommended for its greater use within healthcare [34]. We apply an ethnographic approach to access the beliefs and situated knowledge that govern the healthcare practices that constitute the initial and revised theories, and the experiences of their implementation, thereby also making visible potential antagonisms that are navigated by the participants during the process of the research project [35].

## Methods and analysis

### Project design

We report in this study protocol the design of a three-year project including both qualitative as well as quantitative research methods used for several studies. The project has been approved by the Swedish Ethical Review Authority (Dnr 2020-00659). An informed consent form will be read and signed by all study participants. Findings will be published and presented at conferences. Trial registration number; NTC04336553.

### Patient and public involvement

Prior to the launch of the model for Social Prescribing in Sweden, in preparation of the intervention, several meetings were held with stakeholders, representatives from patients-groups in the field of the older population together with health professionals and representatives from the research group. The purpose of these meetings was for the researchers to facilitate co-creation and out-line the potential content of the social prescribing intervention in Sweden. By gathering opinions from the community along with the target group [older adults], modalities of the intervention were elaborated jointly with the primary health- care staff implementing the SPiS and local activity initiatives in the municipality delivering the prescribed social activities.

#### Ethics and dissemination

In accordance with the Declaration of Helsinki [36] every participant will give written, free and informed consent. Potential harms for participants include the possibility of feeling tired, uncomfortable or emotional during self-rated measures. Instructions to participants how to deal with these troubles will be provided by health care professionals. Following a dissemination plan, findings of this project will be published in peer-reviewed journals in the field of healthcare outcomes and community integration. Conferences targeting various audiences, for example, healthcare professionals and community organisations at the local, national and international level, are also planned.

### Phase one: Developing a social prescribing model for the Swedish context and eliciting the programme theory

The focus of phase one is to develop a social prescribing model adapted for the Swedish context and to elicit the programme theory upon which the model is based. In a first step, a literature review will be carried out to get an overview of international suggestions on important components in social prescribing. The second step is guided by the research question which asks ‘what might a culturally adapted social prescribing model look like in the Swedish context, and how and under what circumstances could it potentially reduce loneliness, promote health and improve well-being among older people?’. The focus is to gather stakeholder’s and society’s ideas about:

1. how to adapt the UK model to better fit the Swedish healthcare context and organization;
2. how to address subjective feelings of loneliness within Swedish community dwelling older adults; and
3. what constitutes important mechanisms for why, how and for whom such a programme *could* or *should* work?

Phase one is thus about gaining knowledge of combinations of contextual aspects, components and mechanisms that are identified in the literature and suggested by the participating stakeholders and society as potentially important in developing a Swedish social prescribing intervention. The exploration of knowledge will then serve as a fundament for building the programme theory that is inherent in the social prescribing intervention.

#### Participants, procedures and analysis

Emerging from the temporary and novel translation of the SP model into SPiS, the following steps are to be taken in phase one and the use of methods will consist of the following aspects.

The systematic literature review will rely on the method described by Gough [37]. The results from the literature review will be conducted initially, used to develop an initial programme theory and give guidance for additional qualitative interviews.

Participants for the stakeholder interviews will be representatives from our collaborating partners at: a) the healthcare center, b) the involved community-based activity initiatives, c) local representatives for older adults (65 years old and older), and d) senior citizens. 4 focus-group interviews will be performed [38] to explore and deeper understand the respondents’ perception of important mechanisms on why, how and for whom SP could work and reduce loneliness in older adults. Convenience sampling will be used to invite participants to the interviews, where up to 8 respondents from each category of representatives (a–d) will be included in each focus-group. Two researchers will facilitate each focus-group which will be audio-recorded, transcribed and analysed using content analysis [39].

In addition, in order to explore and deepen the understanding of perceptions from the society about subjective feelings of loneliness within community dwelling older adults and how to address it, data will be collected using the method of group concept mapping (GCM). GCM is a mixed-method participatory approach that aims to facilitate the understanding of complex phenomena [in this case loneliness], reveal their structures and discover new meaning [40,41]. The participatory approach invites the participants to identify, organize and prioritize ideas in relation to a specific topic, in this case loneliness. Thereafter, the ideas are categorised and multivariate analyses are conducted that include two-dimensional multidimensional scaling [42], and the result presented quantitively. The results are interpreted using a structured group interpretation process, validated and further discussed by the participants and the research-group. Different clusters of people representing society i.e., practitioners, students, community workers and senior citizens will be invited to participate in this online study.

Summing up, the data generated in phase one will build the first programme theory of SPiS and will be tested in the subsequent phases.

### Phase two: Conducting and following up on the SPiS implementation and testing the programme theory

Based on the elicited programme theory and the adapted model developed during phase one, the collaborating partners will implement the SPiS in a regular primary healthcare center over a 10-month test period. The aim of phase two is to capture how the practitioners and older adults experienced the programme, as well as to identify facilitators and barriers for implementation. By collecting qualitative information about the participants’ experiences of the intervention and about its impact or outcomes, important contextual factors and potential mechanisms from different sources will be revealed. The focus of this phase is to explore older adults’ experiences of being prescribed social activities with respect to what works and why, and health practitioner’s expectations and perceptions of SPiS through exploring beliefs about, and experiences of what hindered or facilitated a successful delivery of SPiS. The phase is guided by an interest in the thoughts and feelings of practitioners and older adults about the social prescribing model and by an assessment of its potential effects.

#### Participants, procedure and analysis

For this phase of the project, we will invite and include older adults, 65 years old and older, who are considered to be at risk of loneliness, and who the primary healthcare center has identified as needing social prescribing. Older adults having extensive cognitive decline, or associated serious problems with decision-making and/or communication will be excluded from the interview studies for ethical reasons.

In order to gather the participating practitioners’ and older adults’ experiences of SPiS, repeated interviews will be conducted. Interviews will focus on both experiences of prescribing or having had social activities prescribed and the collection of information on contextual factors. Focus group methodology will be used among the participants been prescribed social activities. Data will be analyzed thematically [43] and guided by the realist evaluation framework.

In addition, the focus of the interviews with practitioners aims to explore their experiences of fidelity, context and mechanisms, moderating factors, as well as barriers in the procedure and will be collected through repeated individual interviews. The interviews will be analyzed ethnographically, in order to capture the cultural notions underpinning the development of SPiS, experiences of a pandemic time and understandings of trying out SPiS. Furthermore, the suggestions for improving SPiS will also be considered. Questions about how notions of the meaning of loneliness, the needs of older adults, and the significance of ‘Swedishness’ might impact the design of a social prescribing model in a Swedish context might here become relevant, as might questions of gender, ethnicity and geographic space [to name just a few possibilities].

### Phase three: Refining the programme theory into a middle-range theory and provide directions for up-scaling the intervention

In this third phase, as guided by the realist evaluation methodology [27], focus will be on ***What works*** in the social prescribing model to reduce loneliness, promote health and improve well-being of older adults, ***for whom, in what conditions*** and ***why***? In conclusion, by posing the previously developed knowledge concerning the SPiS with the knowledge gained in this final phase, in-depth knowledge will be developed and used to inform the process of refining the theory as well as adjustment of the SPiS programme.

#### Participants, procedure and analysis

The collaborating healthcare center has estimated their patient flow of older adults (65 years and older) to be approximately 1000/year. Based on the prevalence of loneliness in this age group [3], at least between 170–400 older adults could probably be identified and potentially recruited to the project during the 10-month test period. The older adults will be invited to participate in the study by the healthcare practitioners at the meeting for social prescribing. Self-reported data related to loneliness, general health and well-being will be collected, as self-administrated measures at the time of referral [baseline] and as a follow-up six months after engagement in their prescribed social activities. The patients will be provided with questionnaires from the primary health care staff at a personal meeting, after giving informed consent. The persons are instructed to return them after completion before engaging in the prescribed social activities. At the six months’ follow-up the participants will be provided with the same set of outcome measures by the staff to be self-administered and returned after completion.

Besides the pre- and post-test collection of data, a selection of participants will be followed more closely with repeated points of measures using the same instruments as described. 5–10 older adults will be followed more closely [every months during 6 months] using both telephone and personal contact. The selection of the participants will rely on the scores from baseline measures of the primary outcome measure, UCLA loneliness scale, to support the inclusion of diverse feelings of loneliness.

Primary outcome

**a)** The UCLA loneliness scale is preferable for studying changes in loneliness interventions, using this scale, participants’ self-reported experiences of loneliness will be measured [44]. The measurement includes 20 statements, rated on a four-step scale from never to always. The given scores are summarized into a total score, ranging from 20–80. The measurement has been used on groups of older people [44], and frequently in intervention studies [45]. The UCLA loneliness scale [44] measures participants’ self-reported experiences of loneliness. The measurement includes 20 statements, rated on a four-step scale ranging from never to always.

Secondary outcomes

**b)** Self-rated general health will be assessed using the visual analogue scale (VAS) from the EQ-5D scale [46]. The VAS is a psychometric response scale which measures subjective characteristics or experiences in a qualitative way. When responding to the VAS item in this project, the respondents specify their level of agreement to the statement “in general how do you experience your health?” by indicating a position along a continuous line between the two end points, “best possible health and worst possible health”.
**c)** Short Form Health Survey Swedish version (SF-12) [47], will be used to capture the overall subjective health status. The SF-12 is a questionnaire that covers physical health and mental well-being. SF-12, developed from SF-36, has established validity and reliability for use in older populations [48].
**d)** Leisure engagement will be measured using the Modified NPS interest checklist (MNPS), which covers 20 leisure activities. For each activity, the participants answer if they are interested in the activity, perform the activity, want to perform it, and if the activity is important for their well-being. The MNPS has been used to evaluate leisure engagement among older people [49,50].
**e)** The GDS-15, depression scale is used to identify symptoms of depression in older adults [51]. The GDS consists of 15 self-rated questions that will assess the level of enjoyment, interest social interaction and more among the older adults. In order to screen for symptoms of depression rather than factors associated with aging, the GDS focuses specifically on psychiatric symptoms rather than somatic.
**f)** Social participation and social support will be measured with a questionnaire, targeting issues related to social living conditions and the extent one participates in social activities in different contexts.

The gathered quantitative data will be analyzed descriptively using Statistical Package for Social Sciences. Descriptive statistics, as well as parametric and non-parametric statistical analysis i.e., Wilcoxon-signed rank test or paired t-test, will be used to detect changes within groups of older adults in the outcome variables.

Finally, the overall quantitative and qualitative data will be brought together using explanatory retroductive inference [52]. This means that the quantitative outcomes will be explained through the mechanisms and contextual factors identified qualitatively to develop a middle-range theory. The SPiS will in this final phase become both theoretically as well as empirically underpinned to explain what combination of components and contextual contingencies have the potential to counteract subjective feelings of loneliness, improve health and well-being among older individuals. Moreover, this final step in the conclusive analysis moreover opens up for further investigation as the cyclist methodology used in this project generates new inquiries about testing this refined programme theory in future studies that focus on *what* in the intervention is working, *for whom*, in *what circumstances*, in *what respects*, and *why* [27].

## Discussion

SPiS lays the foundation for strategic collaboration emerging from shared responsibilities for social interventions among already existing arenas and stakeholders in the welfare state.

As such, this project has the unique position of providing initial knowledge how to close the gap between primary care and societal initiative regarding how engagement in tailored local community social activities might reduce loneliness, promote health and improve well-being among older adults in Sweden.

Qualitative and quantitative data along with subsequent analysis methods will be collected and utilized, something which is known to be challenging [26] in order to measure the achievement of the aim and to grasp the complexity and potential impact of SPiS. Correspondingly, in this pandemic time of Covid-19, social distancing i.e., physical distancing, increases the urgent need for action as people at risk of loneliness might even be lonelier when whole societies close. If equity in health is to be fulfilled this demands structural changes [2] and that provide alternative interventions in times of crisis.

The application of an ethnographic approach to culturally adapt the translated model of social prescribing into a Swedish context is of principal importance. The result from the contextual fit of the programme will not only serve as a facilitator for the model of SpiS in itself, but it might also contribute to enrich knowledge that answers *what* in the intervention is working, *for whom*, in *what circumstances*, in *what respects*, and *why*. As such, this current evaluation of SPiS goes beyond the unidimensional question “*Is it working?*” and it might additionally cope with the criticisms of inconsistency and ambiguity that previous evaluations of social prescribing models have had to endure [19].

On the other hand, important work of developing, implementing and evaluating complex interventions such as SPiS, can only benefit from the availability of a more robust evidence base [16]. Therefore, limitations of this project derives mainly from the methods initially chosen to evaluate the project. At a later stage, it is crucial to apply methods for systematic evaluations, including control groups, with validated measures of the effect from SPiS on important outcome measures such as loneliness, health, well-being, costs and care consumption.

## Conclusion

Building bridges for co-creative integrated care programmes in primary healthcare and local community needs to be a priority [25] for the welfare state. Developing, implementing and evaluating such a complex program needs systematic and close evaluation. Applying the described battery of complex evaluation methodology also positions the basis for potential up-scaling and further implementation and evaluation of SPiS with systematic effect evaluations in a larger context that might involve even more innovative collaborations among care providers, settings and time.

## Strengths and limitations of the research project

- This project has the potential to develop important knowledge regarding how to prevent and/or reduce an urgent public health issue, loneliness among older adults, by means of social prescribing in a Swedish context.
- This current evaluation methods of Social prescribing in Sweden, guided by the use of realist evaluation, goes beyond the unidimensional question “*Is it working?*” and provide knowledge regarding *what* in the intervention that works, *for whom, under what circumstances* and *why* and might additionally cope with the criticisms of inconsistency and ambiguity that previous evaluations of social prescribing models have had to endure.
- Knowledge gained from this project will also not only offer insight into how integrated care might be facilitated by the collaboration between primary healthcare and community activity initiatives on how to work together targeting loneliness, but also raise our understanding of the potential of such a service.
